# Accuracy of Assessment of Eligibility for Early Medical Abortion by Community Health Workers in Ethiopia, India and South Africa

**DOI:** 10.1371/journal.pone.0146305

**Published:** 2016-01-05

**Authors:** Heidi Bart Johnston, Bela Ganatra, My Huong Nguyen, Ndema Habib, Mesganaw Fantahun Afework, Jane Harries, Kirti Iyengar, Jennifer Moodley, Hailu Yeneneh Lema, Deborah Constant, Swapnaleen Sen

**Affiliations:** 1 UNDP/UNFPA/UNICEF/WHO/World Bank Special Programme of Research, Development and Research Training in Human Reproduction, Department of Reproductive Health and Research, World Health Organization, Geneva, Switzerland; 2 Swiss Tropical and Public Health Institute, Basel, Switzerland; 3 School of Public Health, College of Health Sciences, Addis Ababa University, Addis Ababa, Ethiopia; 4 Women’s Health Research Unit, School of Public Health and Family Medicine, Health Sciences Faculty, University of Cape Town, Cape Town, South Africa; 5 Action Research & Training for Health, Udaipur, Rajasthan, India; 6 Independent consultant, Addis Ababa, Ethiopia; Karolinska Institutet, SWEDEN

## Abstract

**Objective:**

To assess the accuracy of assessment of eligibility for early medical abortion by community health workers using a simple checklist toolkit.

**Design:**

Diagnostic accuracy study.

**Setting:**

Ethiopia, India and South Africa.

**Methods:**

Two hundred seventeen women in Ethiopia, 258 in India and 236 in South Africa were enrolled into the study. A checklist toolkit to determine eligibility for early medical abortion was validated by comparing results of clinician and community health worker assessment of eligibility using the checklist toolkit with the reference standard exam.

**Results:**

Accuracy was over 90% and the negative likelihood ratio <0.1 at all three sites when used by clinician assessors. Positive likelihood ratios were 4.3 in Ethiopia, 5.8 in India and 6.3 in South Africa. When used by community health workers the overall accuracy of the toolkit was 92% in Ethiopia, 80% in India and 77% in South Africa negative likelihood ratios were 0.08 in Ethiopia, 0.25 in India and 0.22 in South Africa and positive likelihood ratios were 5.9 in Ethiopia and 2.0 in India and South Africa.

**Conclusion:**

The checklist toolkit, as used by clinicians, was excellent at ruling out participants who were not eligible, and moderately effective at ruling in participants who were eligible for medical abortion. Results were promising when used by community health workers particularly in Ethiopia where they had more prior experience with use of diagnostic aids and longer professional training. The checklist toolkit assessments resulted in some participants being wrongly assessed as eligible for medical abortion which is an area of concern. Further research is needed to streamline the components of the tool, explore optimal duration and content of training for community health workers, and test feasibility and acceptability.

## Introduction

Community health workers (CHWs) are increasingly important frontline providers of health information and services in maternal health, contraceptive services, HIV and tuberculosis management and in community level diagnosis of neonatal and childhood illness [1–7].

In many countries the challenges associated with accessing providers of safe abortion care contribute to unsafe abortion and related morbidity and mortality. In 2008 an estimated 22 million pregnancies were terminated unsafely, 98% of which occurred in resource-poor countries [8] where health care worker shortages are most prominent. While the safety and effectiveness of facility-based non-physician providers of early abortion care using vacuum aspiration or medical abortion has been documented [9–18] the supportive roles of community-based providers have received less attention.

Medical abortion in early pregnancy can be provided at the primary care level and on an outpatient basis. Medical contraindications are limited to a few uncommon conditions like chronic or acute adrenal or hepatic failure, inherited porphyria and allergy to mifepristone or misoprostol. Additionally, clinical judgment may be needed for women with long term corticosteroid use, bleeding disorders, severe anemia, pre-existing heart disease or cardiovascular risk factors [19]. Screening for eligibility is usually done by the clinician providing the medical abortion but potentially could be conducted by other health workers in the clinic or even outside of a health facility. However, to date the tools and approaches for this have not been fully developed or validated.

We conducted a prospective clinic-based diagnostic accuracy study with the dual aim of evaluating the validity of a checklist toolkit to assess a woman's eligibility for early medical abortion (≤ 63 days); and assessing the performance of CHWs in using this checklist toolkit.

## Methods

### Study context and participants

The study was conducted in Ethiopia, India and South Africa, countries where medical abortion with mifepristone and misoprostol is legal and available but unsafe abortion remains a serious health issue [20–24] and CHWs are actively involved in providing health care. In Ethiopia health extension workers are deployed at health posts that provide primary health services. In India accredited social health activists (ASHAs) provide basic health care including pregnancy tests, maternal health counselling and referral, and contraceptive counselling, supply and referral at the village-level. In South Africa, where the National Department of Health’s CHW strategy supports CHWs through NGOs [25], such CHWs typically provide counselling and referral for HIV/AIDS and tuberculosis care.

As this was an initial validation study it was done in clinic rather than community settings. Participants were recruited from one government and two non-governmental organization (NGO) clinics in urban Adama and Assela in Ethiopia; at two urban and three rural NGO clinics in Udaipur, Rajasthan in India; and at five urban NGO clinics in Durban and Cape Town in South Africa. All women who presented to the study clinics for an elective abortion during the study period were invited to participate if they were at least 18 years old, willing to provide informed consent, and able to understand the nature of the study, questions, and instructions given by the research assistant. Willingness to participate in the study was documented by participant signatures or thumbprints on consent forms developed specifically for each study site. The study protocol, including consent procedures, was approved by the Ethics Review Committee at the World Health Organization as well as the Ethiopian Public Health Association Internal Scientific and Technical Review Committee on 4 January 2013 (EPHA/OG/575/13), the Institutional Ethics Committee, Action Research & Training for Health, Udaipur India on 27 November 2012, and the University of Cape Town Faculty of Health Sciences Human Research Ethics Committee on 23 November 2011 (HREC REF: 483/2011).

Clinical assessors in both Ethiopia (N = 8) and South Africa (N = 10) were either health officers or nurses with 36–48 months of professional training. Since such providers are not allowed to provide abortion care in India, clinical assessors in that setting were obstetrician-gynecologists (N = 7). CHW assessors in Ethiopia (N = 10) were health extension workers with a minimum of 10 years of basic education and 12 months of public health training; in India (N = 12) they were ASHAs with a minimum of eight years of basic education and 20 days of public health training or village health workers with a minimum of five years of basic education and 28 days of public health education; and in South Africa (N = 7) they were community-based educators with a minimum of 12 years of basic education and 20 days of public health training.

### Study procedures

The checklist toolkit included a urine pregnancy test, a gestational age wheel, and a checklist [S1 Fig] of screening questions related to result of urine pregnancy test, date of last menstrual period, result of gestational age wheel assessment, and seven questions related to possible contraindications to medical abortion (ectopic pregnancy, bleeding disorders, history of inherited porphyria, serious medical illness, long term medication and allergy to medical abortion drugs). The checklist and gestational age wheel were translated into local languages in Ethiopia and India. Additionally, in Ethiopia the gestational age wheel was adapted to reflect the Ethiopian calendar.

CHWs underwent study-specific training of two to four days (training duration was longer in India where CHWs had less prior formal training) in basic reproductive physiology, medical abortion and implementation of the checklist toolkit. Competency was judged on training posttests. Clinician training was limited to review of study methods and tools.

To be screened-in as eligible for medical abortion the study participant had to have a positive pregnancy test, be within 63 days (nine weeks) gestation as calculated with the gestational age wheel and have no positive responses on the screening questions.

Study participants were initially assessed by a CHW and subsequently by the clinician using identical checklists. Results were classified as eligible, ineligible, or labelled inconclusive if assessors were unable to reach a decision based on the checklist information. After clinicians completed the checklist they assessed eligibility using standard clinical procedures including aa bimanual or ultrasound examination to assess gestational age. This final assessment was considered the reference standard exam, and study participants were classified as eligible or ineligible for medical abortion. Clinicians completed the checklist and placed results in an envelope prior to conducting the reference standard exam.

Data collection forms were reviewed for accuracy and completeness by study staff in-country and at the World Health Organization. Data were double-entered into OpenClinica web-based data management system version 3.1.3 (Akaza Research, Waltham, MA, USA). Validation checks and periodic review of missing values, outliers, inconsistencies, and other errors were conducted. All statistical analyses were done using SAS/STAT ® software (SAS Institute. 2011. The SAS system for Windows. Release 9.3. SAS Inst., Cary, NC, USA).

### Study design, sample size and analytical methods

The sample size of 211 participants at each site was calculated with the assumption that of the women seeking care for abortion, 60% would be eligible for medical abortion. This sample size met the objectives of generating a two-sided 90% confidence interval for sensitivity with a width of 15% assuming a sensitivity score close to 60%, and a two-sided 90% confidence interval for specificity with a width of 15%, assuming a specificity score of 80%.

Baseline characteristics for participants presenting for medical abortion were described, as were characteristics of participating CHWs and clinicians.

We compared results of checklist assessments by clinicians and by CHWs with results of the reference standard assessment by computing statistics for overall accuracy, sensitivity, specificity, positive and negative predictive values, and positive and negative likelihood ratios.

Study recruitment took place between November 2012–February 2014. Project implementation in each country was timed according to partner availability and project approvals.

## Results

CHWs in each country scored similarly on training posttests which measured recall of the didactic material presented in the training (Ethiopia mean: 88% (SD 0·17), India mean: 82% (SD 0·25), South Africa mean: 89% (SD 0·18)).

**Fig 1:**
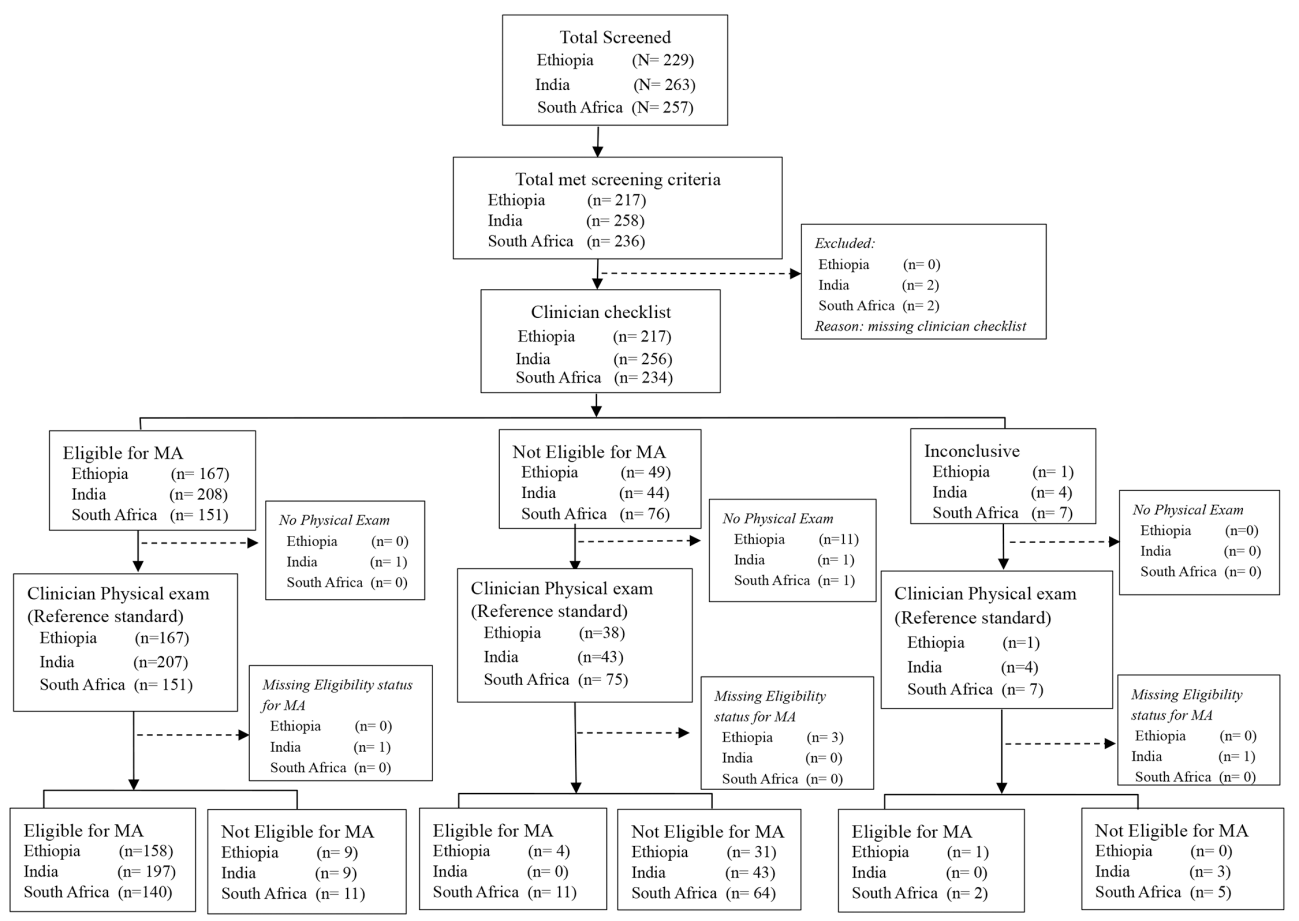
Eligibility for early medical abortion: Assessment of validity of clinician use of eligibility checklist toolkit vs clinician reference standard physical exam.

Ninety-five percent (n = 217) of women coming to the study sites in Ethiopia agreed to participate, 98% in India (n = 258), and 92% in South Africa (n = 236) (Fig 1). Most were in the 20–29 year age group (Table 1). In n India half of participants had no formal education; Sixty percent of participants in Ethiopia and 98% in South Africa were educated beyond class nine.

**Table 1:** Background characteristics of study participants, by country.

	EthiopiaN = 217	IndiaN = 258	South AfricaN = 236	p-value
	n (%)	n (%)	n (%)	
**Age**				
≤ 19	41 (19)	19 (7)	18 (8)	< 0.0001
20–29	154 (17)	146 (57)	149 (63)	
30–39	22 (10)	86 (33)	66 (28)	
≥ 40	0 (0)	7 (3)	3 (1)	
Missing/no answer				
**Education level (years)**				
No formal education	12 (6)	132 (51)	0 (0)	< 0.0001
1–5	19 (9)	41 (16)	0 (0)	
6–8	56 (26)	38 (15)	3 (1)	
9–12	95 (44)	29 (11)	137 (58)	
>12	35 (16)	18 (7)	96 (40)	
Missing/no answer				
**Previous pregnancies**				
0	136 (63)	19 (7)	53 (23)	< 0.0001
1–2	57 (26)	90 (35)	136 (58)	
3–4	12 (6)	106 (41)	42 (18)	
5+	12 (6)	43 (17)	5 (2)	
Missing/no answer				

Reference standard exams were conducted on 206 women in Ethiopia (three forms were missing), 254 women in India (two forms were missing) and 233 women in South Africa. Of these, 163 women (80%) were found to be eligible for medical abortion according to the reference standard exam in Ethiopia, 197 in India (78%) and 153 (66%) in South Africa (Fig 1). Cases whose status was determined inconclusive in the checklist assessment and those with reference standard exam eligibility status missing were excluded from the analysis.

The most common reason for women being determined as ineligible for medical abortion in the reference standard exam was pregnancy >63 days. This was the reason in 63% of the ineligible cases in Ethiopia (25/40); 64% of the ineligible cases in India (35/55) and 89% of the ineligible cases in South Africa (71/80). In Ethiopia 14, India eight, and South Africa two study participants were found to be non-pregnant in the reference standard exam. In India five and in South Africa two study participants required further assessment even after the reference exam. In India three women had already started taking medical abortion tablets prior to coming to the clinic. One study participant in South Africa was suspected to have an ectopic pregnancy; one in Ethiopia was suspected to have a bleeding disorder; four in India and four in South Africa were labelled ineligible for miscellaneous clinical reasons.

### Checklist toolkit as used by clinicians

Accuracy of the checklist toolkit was over 90% and negative likelihood ratios were less than 0.1 at all three sites. Positive likelihood ratios were 4.3 in Ethiopia, 5.8 in India and 6.3 in South Africa (Table 2A).

**Table 2:** Diagnostic test statistics, by country.

	2a. Clinician tool validity (Clinician use of checklist toolkit vs clinician physical exam)						2b. CHW tool validity (CHW use of checklist toolkit vs clinician physical exam)					
Diagnostic test statistic	Ethiopian_CT_ / N_CE_		Indian_CT_ / N_CE_		South African_CT_ / N_CE_		Ethiopian_CHW_ / N_CE_		Indian_CHW_ / N_CE_		South African_CHW_ /N_CE_	
Accuracy (% of all cases correctly identified)	189/202	93.6%	240/249	96.4%	204/226	90.3%	179/195	91.8%	199/250	79.6%	173/225	76.9%
Sensitivity (95% CI)	158/162	97.5(93.8, 99.3))	197/197	100.0 (98.1, 100.0)	140/151	92.7 (87.3, 96.3)	147/157	93.6(88.6, 96.9)	168/196	85.7 (80.0, 90.3)	128/146	87.7(81.2, 92.5)
Specificity (95% CI)	31/40	77.5 (61.6, 89.2	43/52	82.7 (69.7, 91.8)	64/75	85.3 (75.3, 92.4)	32/38	84.2 (68.8, 94.0))	31/54	57.4 (43.2, 70.8)	45/79	57.0 (45.3, 68.1)
Predictive Value (+)	158/167	94.6(90.2, 97.5))	197/206	95.6 (91.9, 98.0)	140/151	92.7 (87.3, 96.3)	147/153	96.1 (91.7, 98.6)	168/191	88.0 (82.5, 92.2)	128/162	79.0 (71.9, 85.0)
Predictive Value (-)	31/35	88.6(73.3, 96.8)	43/43	100.0(91.8, 100.0)	64/75	85.3 (75.3, 92.4)	32/42	76.2 (60.6, 88.0)	31/59	52.5 (39.1, 65.7)	45/63	71.4 (58.7, 82.1))
Likelihood ratio (+)	-	4.3	-	5.8	-	6.3	-	5.9	-	2.0	-	2.0
Likelihood ratio (-)	-	0.03	-	0.0	-	0.09	-	0.08	-	0.25		0.22

n_CT_ = cases identified by clinician using checklist

n_CHW_ = cases identified by CHW using checklist

N_CE_ = cases identified by clinician physical exam (reference standard)

Clinician checklist assessments of pregnancy duration as ≤63 days or >63 days matched the reference standard in 96% of cases in Ethiopia, 88% in India and 95% in South Africa. Study participants were incorrectly assessed as ≤63 days by clinicians with the checklist,in eight cases in Ethiopia (five of these were between 63-≤70 days, two were 70–84 days, and one 143 days), 17 cases in India (nine were between 63-≤70 days, six were 70–84 days, and two 105 days); and seven cases in South Africa (one was between 63-≤70 days, four were 70–84 days, one 91 days and one 112 days).

In a small number of cases (one in Ethiopia, four in India, seven in South Africa), clinician assessors could not reach a decision based on the eligibility checklist, usually because of uncertainty of the date of last menstrual period.

The one study participant with a suspected ectopic pregnancy was not identified by the clinician using the checklist toolkit.

### Checklist toolkit as used by community health workers

Accuracy of CHW use of the checklist toolkit was 92% in Ethiopia, 80% in India and 77% in South Africa. The positive likelihood ratio was 5.9 in Ethiopia and 2.0 in both India and South Africa. The negative likelihood ratio was 0.08 in Ethiopia, 0.25 in India and 0.22 in South Africa (Table 2B).

**Fig 2:**
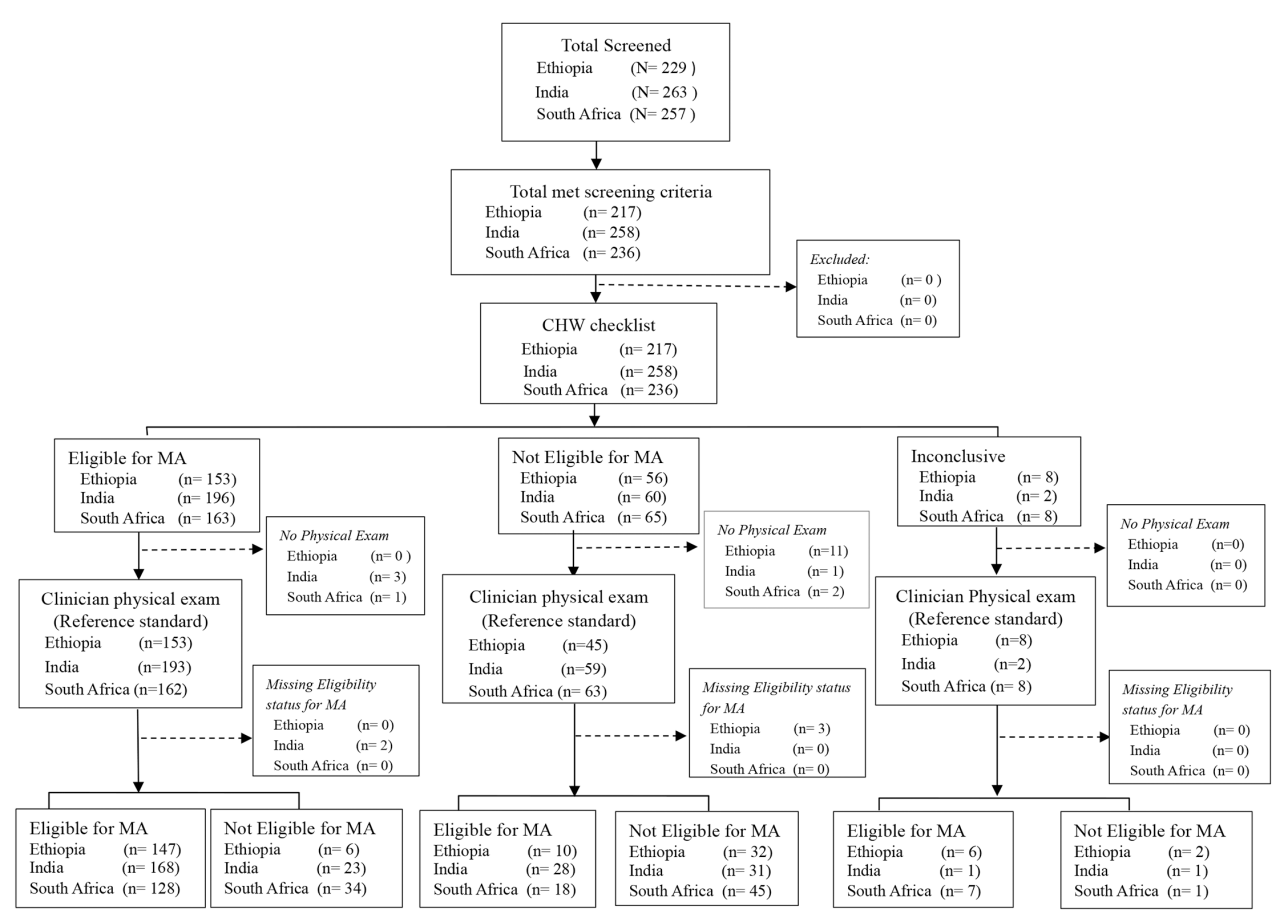
Eligibility for early medical abortion: Assessment of validity of CHW use of checklist toolkit vs clinician reference standard physical exam.

CHWs did not reach a conclusion using the eligibility checklist toolkit for eight women in Ethiopia and South Africa and two in India (Fig 2), usually because of uncertain date of last menstrual period.

CHW categorization of duration of gestation was in agreement with the reference standard in 95% of cases in Ethiopia, 83% in India, and 90% in South Africa. Study participants were incorrectly assessed as ≤63 days by CHWs with the checklist toolkit in six cases in Ethiopia, 29 cases in India and 19 cases in South Africa. In Ethiopia five of these six cases were assessed in the clinician physical exam as 63-≤70 days and one 126 days; in India 11 of the 29 cases were 63-≤70 days, four 77 days, six 84 days, seven between 98–126 days, and one 168 days; and in South Africa six of the 19 cases were 63-≤70 days, five 77 days, four 84 days, three between 91–112 days and one 168 days.

The study participant with a suspected ectopic pregnancy was not identified by the CHW with the checklist toolkit.

## Discussion

This is the first study on CHW use of a checklist toolkit to assess women’s eligibility for medical abortion.

Clinician use of the toolkit yielded negative likelihood ratios of <0.1 in all three countries. Results were similar with Ethiopian CHW assessors, but more modest in India and South Africa. A negative likelihood ratio of <0.1 is a good indicator that the likelihood of a woman who is eligible for medical abortion being wrongly classified as ineligible is low.

However, clinically, it is even more important to ensure that women who are over the gestational age cut off or have other contraindications are not incorrectly labelled as eligible. Results were more modest in this regard with positive likelihood ratios of 4–6 with clinician use of the checklist. With CHWs using the checklist, positive likelihood ratios were modest in Ethiopia but less so in India and South Africa.

Mifepristone and misoprostol are effective even beyond 63 days and recent research suggests similar efficacy using the current regimens to 70 days [26–27]. However a small number (n = 17) of study participants assessed as ≤63 days by clinicians using the checklist toolkit were >70 days gestation according to physical exam results, which is of concern. Women’s self-recall of days since most recent menstrual period is a reasonable proxy for gestational age assessment especially at <63 days [28–29]. Yet in this study, not all participants were able to provide an accurate first date of their last menstrual period and this may have resulted in these discrepancies.

Additionally, at all three sites, CHWs made errors in the use of the gestational age wheel and the recording of its results. This signifies the need for further research in identifying optimum duration and content of training in order to improve the accuracy of CHW assessments.

Overall, the accuracy of CHW checklist toolkit results was higher in Ethiopia than in India and South Africa. This may have been because health extension workers in Ethiopia had higher levels of pre study training and more experience with use of diagnostic aids. As with any new technique, there is a learning curve and results may have improved in India and South Africa over time; the study design however did not allow us to test for this.

The one woman suspected to have an ectopic pregnancy on the reference standard exam was not identified as such through the checklist toolkit. Ectopic pregnancy is rare and even if a woman with an ectopic pregnancy receives medical abortion, it would be ineffective but it would not worsen the condition. Nevertheless, this represents a missed opportunity for early detection. Further iterations of the toolkit could refine screening questions relating to identifying a possible ectopic pregnancy. Very few women were identified as having other medical contraindications which is not surprising given that absolute contraindications to medical abortion are few and the conditions they represent are rare.

One study limitation is that the same clinician who completed the checklist toolkit assessment conducted the reference standard physical exam. We emphasized to clinicians that checklist results must not be altered based on results of the reference standard physical exam but the possibility that this occurred in some instances cannot be ruled out.

The results show that, as used by clinicians and by CHWs in Ethiopia, the checklist toolkit is reasonably accurate and works well for ruling out ineligible cases. It is less effective in ensuring that women are not incorrectly ruled in to receive medical abortion when they should not be. The current version thus cannot replace a second level clinical assessment of eligibility but could be used as a community level screening tool to facilitate appropriate referrals and timely triage. That CHWs with minimal schooling or prior experience with diagnostic aids were also able to use the toolkit, suggests the potential of developing this approach further.

## Conclusion

Simple tools to help community health workers assess eligibility for early medical abortion is a promising approach that has the potential to decentralize access to safe care and overcome shortages of physician providers. Further research is needed to refine the checklist toolkit and explore the feasibility and acceptability within community settings.

## Supporting Information

S1 Fig Medical abortion eligibility checklist tool. (PDF)
