# Supplementary material for: Accuracy of Assessment of Eligibility for Early Medical Abortion by Community Health Workers in Ethiopia, India and South Africa
Source: PLoS One. 2016 Jan 5;11(1):e0146305. doi: 10.1371/journal.pone.0146305 (PMC4701452; doi:10.1371/journal.pone.0146305)
Supplement: S1 Fig — (PDF) [file pone.0146305.s001.pdf]

World Health Organization	A65853 - Assessment of eligibility and follow-up care for early medical abortion<br>ASSESSMENT OF ELIGIBILITY (CHW)	AE1<br>page 1/2<br>DD/Mon/YYYY

Center number ☐☐☐☐ Screening number ☐☐☐☐ Subject number ☐☐☐

Please insert the number relating to the response into the adjacent box.

(Questions 1-3 to be completed by the Research Assistant)

1. Facility Name: ☐
N1 = Facility 1
N2 = Facility 2
N3 = Facility 3

2. Assessor ID: ☐☐

3. Date of assesment:

Day		Month			Year			

URINE PREGNANCY TEST

Please put an "X" in the box relating to the response.

4. Result of urine pregnancy test: 1 ☐ 2 ☐ 3 ☐
1 = Negative
2 = Positive
3 = Indeterminate

If "Negative" the woman is not eligible for medical abortion. You do not need to continue.

LAST MENSTRUAL PERIOD

5. Does the woman know the date of her last menstrual period? N ☐ Y ☐ NS ☐
N = No
Y = Yes
NS = Not sure

6. What was the first day of the woman's last menstrual period?

Day		Month			Year			

(If the woman does not remember exact date, ask for an approximate date)

USE THE PREGNANCY WHEEL

7. Where does today's date fall on the pregnancy wheel? 1 ☐ 2 ☐
1 = Green area
2 = Red area

If the arrow is in the red area, she might not be eligible for medical abortion.

If today's date falls in the green area, then record the duration of pregnancy:

a) Weeks ☐ b) Days ☐

HEALTH STATUS CHECKLIST

Ask the woman the following questions.

Please put an "X" in the box relating to the response.
N = No
Y = Yes
NS = Not sure

	N	Y	NS
8. Did you have unusual bleeding during your last period?	☐	☐	☐
9. Do you have bleeding problem? *(Very heavy bleeding after childbirth or miscarriage, cuts that don't stop bleeding, or frequent severe nosebleeds)*	☐	☐	☐
10. Do you have a history of inherited porphyria? *(Disease that runs in your family where you are very allergic to the sun, where you get very ill with vomiting, skin inflammation, confusion and muscle weakness. NOT just SUNBURN)*	☐	☐	☐
11. Have you ever had a pregnancy in your tubes, i.e. an ectopic pregnancy?	☐	☐	☐
12 or medical conditions?	☐	☐	☐
13. Are you taking any prescribed medicines?	☐	☐	☐
14. Do you have a loop /IUD /IUCD now? *(Copper T, Mirena)*	☐	☐	☐
15. Do you have pain in your lower abdomen today?	☐	☐	☐
16. Do you have any vaginal bleeding today?	☐	☐	☐

ASSESSMENT OF ELIGIBILITY (CHW)

Center number ☐☐☐☐ Screening number ☐☐☐☐ Subject number ☐☐☐

Ask the woman the following questions (continued)
N= No
Y = Yes
NS = Not sure

	N	Y	NS
17. Have you ever had an allergic reaction to medical abortion pills?	☐	☐	☐

Thank you for participating in our study, please wait a moment whilst I check that I have asked you all the questions.

Assessor: Please do not read the following out loud.

ELIGIBILITY ASSESSMENT

If there is no tick in the pink box or shaded box, the woman is eligible for medical abortion today.

If there is at least one tick in the pink box or shaded box, the woman might not be eligible for medical abortion.

18. Do you think this woman is eligible for medical abortion today? ☐
(Please insert the number relating to your assessment in the box)
1 = No
2 = Yes
3 = Not sure

a) If "Not eligible" or "Not sure", please explain WHY?

____

____

____

____

Please ensure that all responses have been completed and date and sign the form.

Then please send the woman to the Research Assistant with the completed form.

Assessor's Name: ***Signature:***

Research Assistant's Name: ***Signature:***

Date:

Day		Month			Year			

Data Entry Operator's signature and date:

1st DE: ____________________

2nd DE: ____________________
